# Sex-dependent differences in mitochondrial protein acetylation in metabolic condition, oxidative stress, vascular dysfunction, hypertension, and cardiovascular disease

**DOI:** 10.1042/CS20250226

**Published:** 2026-05-29

**Authors:** Anna Dikalova, Sergey Dikalov

**Affiliations:** Vanderbilt University Medical Center, Nashville, Tennessee, U.S.A.

**Keywords:** acetylation, antioxidant, hypertension, mitochondria, oxidative stress, sex

## Abstract

One half of adults have hypertension, which is a major risk factor for stroke, myocardial infarction, heart failure, and vascular dementia. There is an urgent need for new therapies, particularly for women with hypertension. Hypertension affects women in all phases of life; however, the hypertension rate increases in women much more steeply, and hypertensive vascular and kidney damage is significantly higher in women. Despite great burden, only 1 in 4 patients have their blood pressure under control. Hypertension accounts for 1 in 5 deaths among American women, posing a greater burden for women than men. Meanwhile, female-specific aspects of hypertension are poorly understood, and women or female-specific risk factors are understudied in basic, clinical, and population research and hypertension guidelines. Understanding these mechanisms can help to develop new therapies. Endothelial dysfunction has a profound prognostic implication predicting adverse cardiovascular events. We suggest that female antihypertensive protection is critically dependent on mitochondrial pathways preserving endothelial function. Metabolic disorders and oxidative stress contribute to the pathogenesis of these conditions, which are linked to mitochondrial dysfunction. Proteomic studies showed higher expression of mitochondrial fatty acid oxidation and antioxidant enzymes in females, and oxidative damage is lower in females compared with males. Meanwhile, the actual activity of these mitochondrial metabolic and antioxidant enzymes is regulated by acetylation, but sex-specific differences in mitochondrial acetylation in vascular disease have not been studied. In the present review, we will discuss potential sex differences in mitochondrial protein acetylation and its implications in metabolic conditions, oxidative stress, vascular dysfunction, hypertension, and cardiovascular disease.

## Introduction

**Figure 1:**
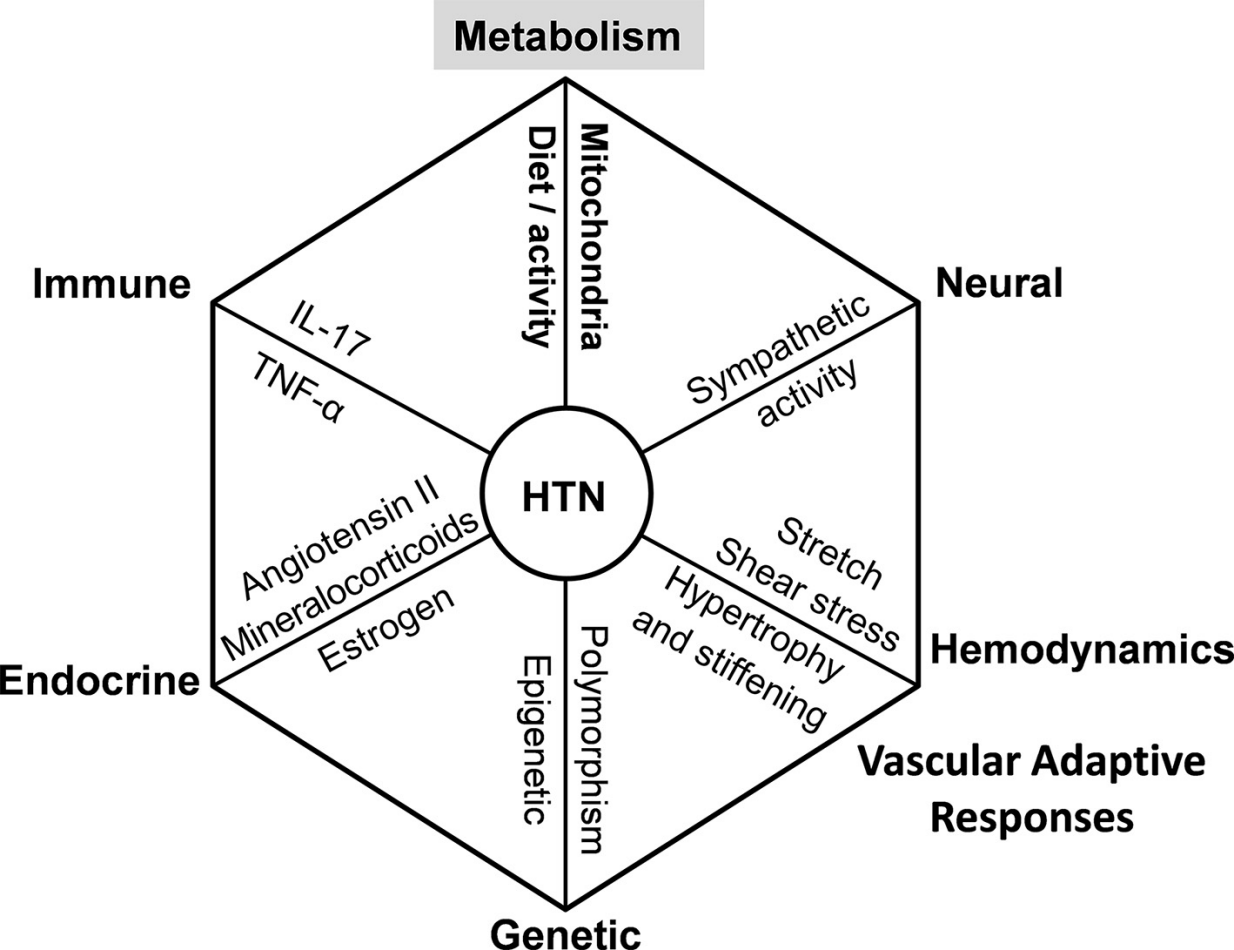
Multiple causes of hypertension and cardiovascular disease. Hypertension (HTN) is a multifactorial disorder that is promoted by multiple pathways [9]. Dysregulation of metabolic, immune, endocrine, neural, genetic, and epigenetic pathways and hemodynamics and vascular adaptive responses contribute to the development of hypertension and cardiovascular disease. This figure is modified from our previous publication [10].

Endothelial dysfunction plays a key role in the pathogenesis of hypertension [1], and half of adults in the United States have hypertension, which represents a main risk factor for cardiovascular disease, killing 700,000 people every year in the US alone [2]. Hypertension is a multifactorial disorder (Figure 1) involving perturbations of vasculature, kidney, genetics, central nervous system, endocrine, and immune systems [3]. Recent studies suggest that metabolic conditions are linked to endothelial dysfunction and multi-organ damage, promoting hypertension and cardiovascular disease [4]. It is important to note that all these factors contribute to age-dependent hypertension and cardiovascular disease. Hypertension affects women in all ages, from young adulthood through pregnancy and menopause [5]. It is, however, incorrect to limit the hypertension sex differences to changes in the reproductive cycles, pregnancy, contraceptives, or hormone replacement therapies. The differences in multiple systems including endocrine and metabolic pathways may reduce the prevalence of hypertension in early adulthood among women compared with men [6], but with aging, the hypertension rate increases in women much more steeply, and hypertensive vascular and kidney damage is significantly higher in women [7,8]. Gender-specific aspects of hypertension are still poorly understood. In the present work, we will discuss the potential role of mitochondrial protein acetylation and metabolic and oxidative stress pathways in sex differences in vascular dysfunction, hypertension, and cardiovascular disease.

More than one-third of mitochondrial proteins are acetylated, which includes key metabolic and antioxidant enzymes [11]. Acetylation of mitochondrial proteins can be both non-enzymatic and enzymatic, and regulatory lysine residues may be particularly sensitive to acetylation [12]. Acetylation and deacetylation of mitochondrial proteins primarily occur on ε-amino group of lysine residues, which depend on multiple factors: diet, lifestyle, Acetyl-CoA level (acetylation substrate), GCN5L1 acetylase, and Sirt3 deacetylase [13,14]. Other enzymes include deacylase Sirt4 (removing methylglutaryl) and Sirt5 (removing succinyl, malonyl, and glutaryl groups from lysine residues) [15]. NAD^+^-dependent Sirt3 is the only identified mitochondrial deacetylase [16]; however, supplementation of NAD^+^ precursors showed very limited effects [17]. Despite the abundance of mitochondrial protein acetylation, the gender-specific differences in mitochondrial acylation and acetylation in cardiovascular disease are not clear. The sections below provide a discussion of the potential role of differential protein acetylation in homeostatic and pathophysiological pathways.

## The role of metabolic regulations

Mitochondria are essential cellular ‘powerhouses,’ producing the majority of ATP [18]. Interestingly, mitochondria have several sex-specific features. First, female mitochondria are passed exclusively from mother to child, which is the basis of mitochondrial maternal inheritance [19]. Second, female mitochondria are critical for fertility due to high energy demands of oocyte maturation, fertilization, and early embryo development [20]. Third, female mitochondria require higher ‘quality control’ compared with male mitochondria to minimize mitochondrial damage and reduce mitochondrial dysfunction to support fertility and health of the offsprings [21]. It has been suggested that enhanced quality control, lower production of mitochondrial reactive oxygen species, and increased antioxidant activity contribute to a longer lifespan in women compared with men [22].

Most of the cells utilize both mitochondrial oxidative phosphorylation and glycolysis for ATP production. Recent studies showed that vascular cells have balanced utilization of glycolysis and mitochondrial respiration [23]. It is important to note that mitochondrial dysfunction promotes a maladaptive switch to glycolysis, which is detrimental for vascular and endothelial function [24]. Mitochondrial ATP production is critical for the maintenance of endothelial and epithelial barriers, nutrient transport, and cellular phenotype regulations [23,25–27]. Mitochondrial oxidative phosphorylation utilizes multiple substrates, particularly fatty acids, glucose, and amino acids [28]. Meanwhile, fatty acids are the primary source of energy in multiple cells and tissues, including heart, kidney, and vasculature [29], and impairment of mitochondrial fatty acid β-oxidation is particularly detrimental [27,30]. The sex differences in mitochondrial metabolism are largely ignored. Previously, Busija and colleagues performed proteomic studies of cerebral mouse microvessels, showing that 64 proteins had significantly higher expression in female mitochondria compared with male [31]. Interestingly, mitochondrial responses of cerebral arteries to middle cerebral artery occlusion in females are substantially different from responses seen previously in male rats, suggesting the need for specific sex-based therapies [32].

Analysis of female mitochondria showed higher expression of proteins in energy production, membrane structure, antioxidants, and fatty acid oxidation [31]. These data suggest a substantial difference in substrate utilization between men and women mitochondria. Indeed, recent studies showed that females exhibit higher fat oxidation and more mitochondrial metabolic flexibility, while males display higher rates of glycolysis [33]. Female mitochondria prioritize fatty acid oxidation over carbohydrates during exercise, which is supported by increased mitochondrial volume density and fatty acid and lactate oxidative capacity in skeletal muscle fibers [34]. Meanwhile, male metabolism has a higher reliance on carbohydrates and higher rates of glycolysis [33,35].

We suggest that female antihypertensive protection in early adulthood is critically dependent on metabolic mitochondrial pathways, particularly fatty acid oxidation. Indeed, women aging and menopause are accompanied by decreased fatty acid oxidation and detrimental accumulation of free fatty acids and lipids [36]. Sex hormones can promote fatty acid oxidation and reduce triglycerides and cholesterol, therefore diminishing cardiovascular risk [37]; however, this protection is lost with age. Meanwhile, the precise molecular mechanism remains unknown. Impairment of fatty acid oxidation makes females more susceptible to endothelial dysfunction and hypertension due to higher reliance on fatty acid metabolism [38]. This can be mediated by reduced ATP production, pathogenic intracellular accumulation of fatty acids, and mitochondrial dysfunction [39]. Endothelial fatty acid accumulation promotes oxidative stress, increases vascular inflammation [40], and damages endothelial barrier function [27]. Indeed, impaired fatty acid metabolism promotes endothelial dysfunction and hypertension [41], and high-fat diet induces endothelial dysfunction and accelerates atherosclerosis and hypertension [42], indicating that direct exposure of endothelium to high levels of fatty acids is detrimental [43]. Meanwhile, the actual activity of mitochondrial metabolic enzymes is regulated by acetylation [44], but sex-specific differences in mitochondrial acetylation in vascular disease have not been studied.

**Figure 2:**
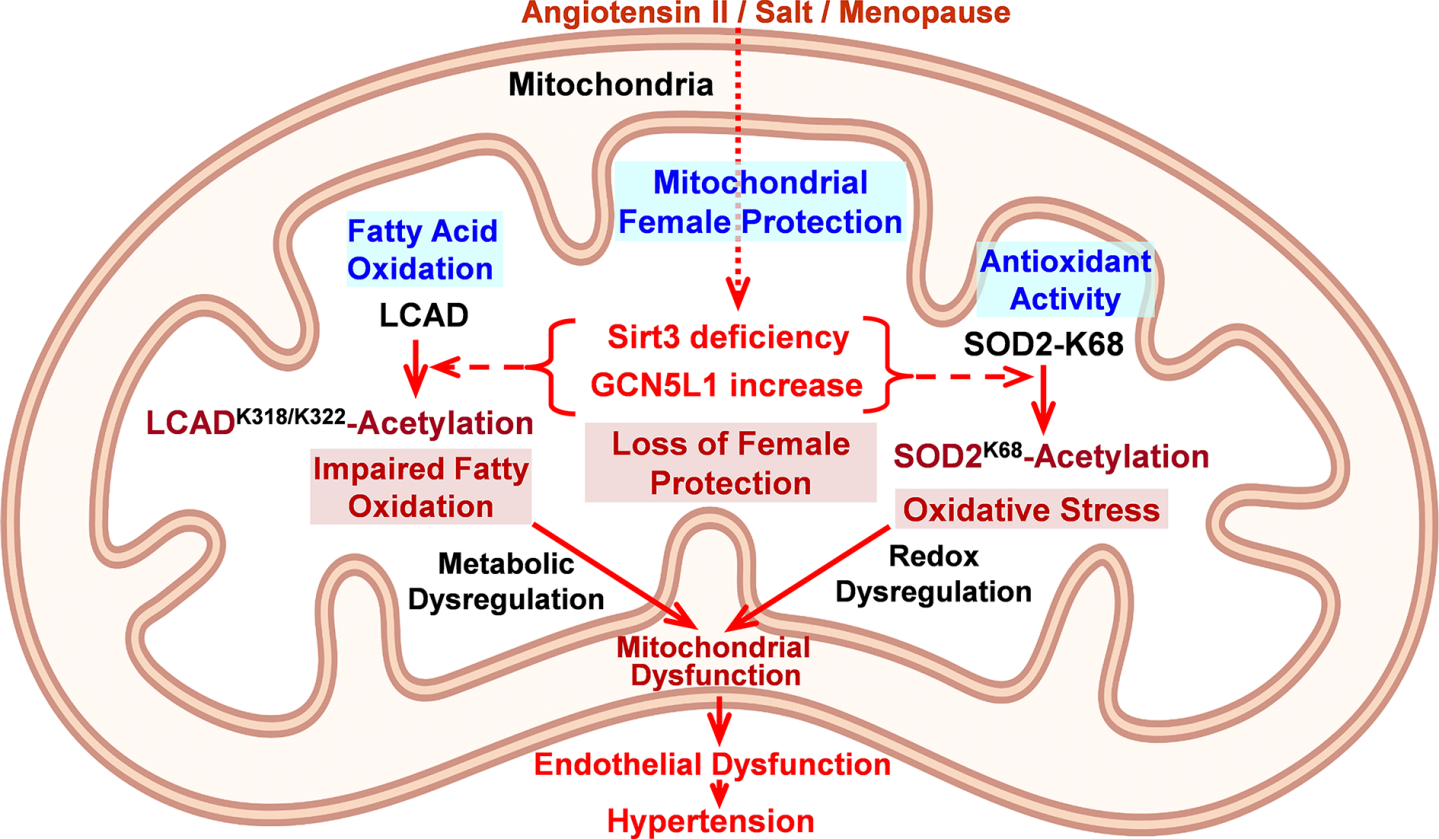
Pathophysiological role of mitochondrial protein acetylation in female cardiovascular health. We propose that female antihypertensive protection in early adulthood is linked to the reduced acetylation of mitochondrial proteins; however, an imbalance between deacetylase Sirt3 and acetyltransferase GCN5L1 results in the acetylation of critical metabolic and antioxidant enzymes such as LCAD and SOD2, which impair fatty acid oxidation and promote oxidative stress, leading to endothelial dysfunction and the loss of female antihypertensive protection.

Multiple cardiovascular risk factors such as high-fat diet, diabetes, smoking, inflammation, and aging promote acetylation of mitochondrial proteins [13]. This can be mediated by the accumulation of acetyl-CoA, non-enzymatic lysine acetylation, acetyltransferase GCN5L1, or a deficit of deacetylase Sirt3 [24]; however, gender differences between these pathways is not clear. We have previously shown increased acetylation of mitochondrial proteins in patients with essential hypertension associated with Sirt3 deficiency, and Sirt3 knockout mice are prone to hypertension [45]. Our recent study showed that hypertension is linked to an imbalance between acetyltransferase GCN5L1 and deacetylase Sirt3 [24]. Sirt3 induces mitochondrial fatty acid oxidation by deacetylation of long-chain acyl coenzyme A dehydrogenase (LCAD) at K318/322 lysine residues [44,46]. Sirt3 also activates key mitochondrial antioxidant enzyme, superoxide dismutase 2 (SOD2), by deacetylation of specific lysine K68 [47–49]. We propose that females have lower LCAD and SOD2 acetylation; however, an imbalance between Sirt3 deacetylase and GCN5L1-mediated acetylation inactivates LCAD and SOD2 (Figure 2), leading to harmful accumulation of long-chain fatty acids and oxidative stress, promoting endothelial dysfunction and hypertension, particularly in females.

It has been suggested that fatty acid oxidation produces acetyl-CoA and therefore promotes protein acetylation [50]. The interplay between fatty acid β-oxidation and protein acetylation is much more complex. First, fatty acid β-oxidation occurs at two sites: mitochondria and peroxisomes [51]. There is a cross-talk between these sites where peroxisomes perform initial oxidation of very-long-chain fatty acids and branched-chain fatty acids into a shorter chain fatty acids for subsequent mitochondrial oxidation [52]. Second, mitochondrial fatty acid β-oxidation is processed by functional fatty acid oxidation-to-electron transport chain multienzyme respirasome complex ensuring that products of one reaction are efficiently passed to the next component without release of acetyl-CoA or other intermediates [50,53]. The resultant acetyl-CoA is passed along to Krebs-cycle for citrate synthesis or immediately transformed to ketone bodies [50]. Third, it was found that peroxisomes rather than mitochondria are the source of mitochondrial protein acetylation [54]. Mitochondria promotes cytoplasmic or nuclei protein acetylation by exporting citrate into cytosol or nucleus where it is transformed into acetyl-CoA [55]. Finaly, metabolic dysfunction, hyperglycemia and high-fat diet lead to pathogenic accumulation of fatty acids, overproduction of acyl-CoA and acetyl-CoA driving deleterious protein hyperacetylation [56].

## The role of oxidative stress

Hypertension is strongly associated with oxidative stress due to an imbalance between antioxidant activity and overproduction of reactive oxygen species (ROS; O_2_^•-^ and H_2_O_2_) by mitochondria, NADPH oxidases, xanthine oxidases, and uncoupled nitric oxide synthase [57–59]. Oxidative stress drives endothelial dysfunction, inflammation, and end-organ damage. Interestingly, there is a redox-dependent regulation of these sources of oxidants, resulting in a feed-forward vicious cycle of oxidative stress [60]. We have previously reported that NADPH oxidases induce mitochondrial ROS production, which in turn increases activity of NADPH oxidases, resulting in mitochondria–NADPH oxidase cross-talk [61], which promotes endothelial dysfunction and development of hypertension [62,63].

**Figure 3:**
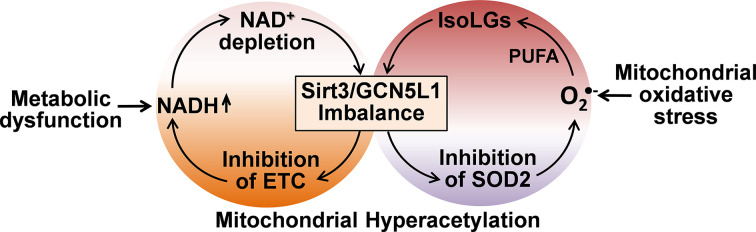
Metabolic dysfunction-oxidative stress crosstalk. The crosstalk between metabolic dysfunction and mitochondrial oxidative stress induces Sirt3/GCN5L1 imbalance driving protein acetylation and mitochondrial dysfunction. PUFA, polyunsaturated fatty acids; IsoLGs, reactive dialdehyde isolevuglandins; ETC, electron transport chain.

Hypertension is associated with both increased production of mitochondrial ROS and inactivation of critical intrinsic antioxidant SOD2 [45,64]. Earlier studies suggested that mitochondrial ROS are ‘by-products’ of oxidative phosphorylation due to ‘spontaneous’ leakage of electrons from the mitochondrial electron transport chain [65]. We think this is an artefact of *in vitro* experiments with isolated mitochondria in air-saturated hyperoxia media (20% oxygen versus 5% *in vivo*) and maximal substrate concentration above Km showing significant superoxide production in the mitochondrial matrix and substantial release of H_2_O_2_ outside of mitochondria [66]. Cell damage, inflammation, and metabolic alterations can induce mitochondrial ROS production by modulation of specific enzymatic functions, and this is not a ‘spontaneous’ leakage of electrons to oxygen as previously suggested. Specific activation of PKCε induces mitochondrial superoxide production via reverse electron transfer to the ubiquinone site of complex I, while blocking the PKCε with a specific peptide inhibitor or targeting reverse electron transfer (malonate or rotenone) prevents overproduction of mitochondrial superoxide and reduces vascular oxidative stress and hypertension [62,63]. SOD2 level was not changed in patients with essential hypertension; however, the SOD2 activity was markedly reduced due to specific acetylation of the highly conserved catalytic center SOD2 lysine 68 [45,67]. SOD2 acetylation is controlled by acetyltransferase GCN5L1 and deacetylase Sirt3, and imbalance between GCN5L1 and Sirt3 (Figure 3) promotes SOD2 acetylation in hypertension [24].

Despite similar expression of SOD2 in males and females [31], the SOD2 activity is higher in females compared with males [68]. This can be due to diminished SOD2 acetylation in female mitochondria. Indeed, the deacetylation mimetic SOD2-K68R mutation reduced angiotensin II-induced hypertension by 24 mmHg in males, while in SOD2-K68R female mice, hypertension was decreased only by 10 mmHg. The potential benefits of increased SOD2 activity can be smaller in females compared with males due to higher basal SOD2 activity in females. We suggest that this antioxidant protection is lacking in female patients with cardiovascular risk factors such as metabolic conditions, salt sensitivity, smoking, menopause, and aging [13,69].

We have developed mitochondria-targeted SOD2 mimetics, which can compensate for SOD2 inactivation including mitoTEMPO, mCP1, and mCP2 [70,71]. Mitochondria-targeted SOD2 mimetics protect mitochondrial respiration, reduce vascular oxidative stress, improve endothelial nitric oxide production, and reduce hypertension [71]. Interestingly, SOD2 acetylation is a redox-dependent process, and it can be reversed and normalized. For example, scavenging of mitochondrial H_2_O_2_ by mitoEbselen and mitochondria-targeted catalase improved Sirt3 activity, rescued SOD2 deacetylation, and reduced vascular oxidative stress and hypertension [45]. These data support the pathophysiological role of SOD2 acetylation in vascular disease and hypertension. Meanwhile, sex-specific role of SOD2 acetylation in female-specific risk factors is understudied in basic, clinical, and population research.

Acetylation of mitochondrial electron transport chain and Krebs cycle enzymes may include complex I–V, isocitrate dehydrogenase, aconitase, and pyruvate dehydrogenase [72]. This not only impairs oxidative phosphorylation but also reduces antioxidant activity due to diminished NADPH production and increases mitochondrial ROS production due to the accumulation of metabolic substrates and overreduction of electron transport chain [56]. The interplay between acetylation of metabolic enzymes and oxidative stress is not well studied.

Mitochondrial oxidative stress leads to lipid peroxidation of polyunsaturated fatty acids such as arachidonic acid and results in the formation of highly toxic lipid peroxidation products, isolevuglandins [73], that cause an imbalance between mitochondrial protein acetylation and deacetylation (Figure 3) [24]. Human hypertension is linked to increased production of isolevuglandins [74], and animal studies showed that blocking mitochondrial isolevuglandins with mitochondria-targeted mito2HOBA improves mitochondrial function, rescues endothelial nitric oxide production and vasorelaxation, and reduces hypertension. It is important to note that isolevuglandins are highly reactive dicarbonyls rapidly making protein adducts that activate dendritic cells, induce T cells promoting cytokine production, vascular alterations, kidney damage, and hypertension [74–76]. Isolevuglandins can represent an important new therapeutic target, particularly considering interaction of oxidative stress and fatty acid accumulation associated with mitochondrial dysfunction described above.

## Inflammation and mitochondria

Although multiple interdependent pathways contribute to hypertension and cardiovascular disease [77], it has become clear that inflammation represents a key node in these pathological conditions [78,79]. Chronic inflammation is both the cause and the consequence of hypertension mediated by activation of T cells, monocytes, macrophages, dendritic cells, B cells, and natural killer cells. Inflammatory pathways are prompted by hormones, salt, mechanical stretch, and metabolic conditions inducing NLRP3 inflammasome, isolevuglandin-adducted peptides, and their processing by the immunoproteasome [78,79]. The critical contribution of inflammation in hypertension and cardiovascular disease has been extensively studied. In the present work, we would like to highlight the potential role of mitochondria and protein acetylation in these conditions. First, mitochondrial damage directly induces NLRP3 inflammasome due to the release of damage-associated molecular patterns (DAMPs) from mitochondria such as mtDNA [80]. Second, mitochondrial ROS induces NLRP3-dependent inflammasome activation [81]. Third, Sirt3 deficiency and acetylation of mitochondrial proteins increase NLRP3 inflammasome and NF-κB activity [67]. Finally, lipid peroxidation product isolevuglandins-peptide adducts are processed by immunoproteasome in dendritic and endothelial cells [76] and presented by murine class I major histocompatibility complex in hypertensive tissue [82] to promote inflammation, vascular dysfunction, and hypertension. Interestingly, these proinflammatory mechanisms are dependent on acetylation of mitochondrial proteins. Depletion of mitochondrial GCN5L1 acetyltransferase markedly reduced production of inflammatory cytokines [83], while mitochondrial deacetylase Sirt3 reduces inflammation and attenuates endotoxin-induced lung injury [84]. This can be mediated by deacetylation/activation of critical mitochondrial antioxidant SOD2 and protection from mitochondrial permeability transition pore opening, inducing cell death. Indeed, we showed that SOD2-K68R [85] and CypD-K166R [24] deacetylation mimetic mice were cytokine-resistant and protected from cytokine-induced endothelial dysfunction and hypertension. Furthermore, genetic overexpression of mitochondrial deacetylase Sirt3 reduces NLRP3 and NF-κB activation, diminished markers of endothelial inflammation VCAM and ICAM, and abolished inflammatory cell infiltration in vascular tissue [67]. Of note, mitochondrial acetylation affects NF-κB and NLRP3 pathways indirectly by increasing production of mitochondrial ROS [86] and mitochondrial damage releasing DAMPs, which activates NLRP3 inflammasomes [87]. These data strongly support the novel role of mitochondria and protein acetylation in inflammatory mechanisms of vascular disease, hypertension, and cardiovascular conditions. It is conceivable that sex differences in mitochondrial quality and protein acetylation such as SOD2 and CypD contribute to the increased rates of hypertensive vascular and kidney damage in women.

## Endocrine dysregulations

It has been shown that estrogen is an important regulator of mitochondrial function, ATP production, mitochondrial biogenesis, and fatty acid metabolism [88,89]. The hormonal changes make postmenopausal women particularly vulnerable to muscle loss and abdominal obesity, which can be affected by a sedentary lifestyle due to changes in energy expenditure and metabolic rate [89]. Animal studies showed that protective effects of estrogen [90] are Sirt3 dependent and are lacking in *Sirt3^−/−^* mice [91], which suggests a potential link between estrogen and mitochondrial acetylation.

Angiotensin II is a critical hormone in the renin–angiotensin–aldosterone system regulating blood pressure and sodium and water retention; however, dysregulation of angiotensin II pathways contributes to hypertension and cardiovascular and kidney disease [92]. In females, the expression of the protective angiotensin type 2 receptor is driven by estrogen, and the type 2/type 1 receptor balance is lost with age and menopause [93,94]. Angiotensin II type 1 receptor has higher expression in males [95], and it can drive activation of redox-dependent NADPH oxidases [63]. Protective angiotensin type 2 receptor is increased in females and promotes mitochondrial biogenesis and increases sirtuins’ activity [96]. These data support the cross-talk between the endocrine system and mitochondria. Additional studies can provide further mechanistic insight and novel pharmacological targets.

## Genetic and epigenetic mechanisms

Genome-wide association studies showed more than 100 variants associated with blood pressure; however, these factors have typically small effect sizes and could explain about 3.5% of blood pressure variability [97]. Sixty-two genes are proposed to have an association with primary hypertension, but only 21 genes have nearly 50% of positive associations [98]. It has been suggested that genetic variants contribute more to high blood pressure in women than in men, leading to higher genetic risk for the development of hypertension in early adulthood [99]. Estrogen receptor-β gene variation is associated with salt-sensitive hypertension in premenopausal women [98], which can potentially include metabolic and mitochondrial effects. The interaction of estrogen decline and genetic factors contributes to accelerated vascular aging [100]. Despite tremendous progress in genetic studies, there are gaps in knowledge about the role of age, sex hormones, genetics, and lifestyles in the development of hypertension and cardiovascular disease.

Recent studies implicate epigenetic modifications in the pathophysiology of essential hypertension [101]. Epigenetics refers to reversible DNA methylation and histone modifications that alter gene expression without changing the DNA sequence. Epigenetic factors are affected by maternal diet, stress, smoking, environmental pollutants, and physical activity [102]. It is important that epigenetic modulation is transmitted to offspring and affect susceptibility to metabolic and cardiovascular diseases for multiple generations [103]. Mitochondrion is key player in epigenetic modulation due to the essential role of mitochondrial metabolic intermediates (acetyl-CoA, NAD^+^, and α-ketoglutarate) for epigenetic enzymes [104]; therefore, mitochondrial metabolism directly affects the epigenetic remodeling, connecting modifiable lifestyle factors with gene expression. This is retrograde signaling where mitochondria send signals to the nucleus to induce changes in gene transcription, which affects cell adaptation and function. As we discussed above, female mitochondrial metabolism is uniquely turned for fatty acid oxidation, which is a major source of acetyl-CoA and other metabolites affecting epigenetics. Dysfunctional mitochondria drive pathogenic epigenetic changes promoting ovarian aging and reducing longevity [105]. Interestingly, targeting mitochondrial function, such as time-restricted eating, exercise, and diet, can improve the epigenetic landscape [106].

## Vascular dysregulations

Vascular disease affects both genders; however, women are more likely to have worse outcomes due to delayed diagnosis and atypical symptoms [107]. Hypertensive vascular damage is much higher in women [7,8], and sex-specific aspects of hypertension are poorly understood [108]. Mitochondrial dysfunction promotes vascular disease due to increased inflammation, oxidative stress, and endothelial dysfunction [109]. Mitochondrial ATP is critical for nitric oxide production and endothelial-dependent relaxation [25], and impaired mitochondrial fatty acid oxidation drives pathogenic endothelial-to-mesenchymal transition [27]. It has been previously suggested that endothelial cells rely on glycolysis [110] instead of mitochondrial respiration, suggesting a minor role of mitochondria in endothelial metabolism. Recent studies demonstrated a balanced utilization of glycolysis and mitochondrial respiration by vascular cells [23], which are normally coupled [111], meaning that glycolysis product pyruvate is utilized by mitochondria. We have shown that mitochondrial dysfunction in hypertension leads to uncoupled glycolysis, i.e., glycolysis is disproportionately increased compared with mitochondrial respiration [24,112]. The increase in vascular glycolysis in hypertension is linked to glycolytic metabolism in endothelium, smooth muscle cells, fibroblasts, and inflammatory cells. Our data show 10-fold increase in endothelial glycolysis in hypertension due to hyperacetylation of mitochondrial proteins, and blocking mitochondrial dysfunction reduces endothelial glycolysis by 50% [24]. The pathophysiological role of glycolytic switches in vascular smooth muscle cells and adventitial fibroblasts [113] promotes aortic aneurysms and vascular fibrosis [114], and inhibition of vascular glycolysis reduces aneurysmal formation and diminishes mortality due to reduced aortic ruptures [115,116]. These data support the novel role of mitochondrial protein acetylation in vascular disease; however, its specific mechanism and sex-specific role remained understudied.

## Central nervous system

Psychological stress, high salt intake, angiotensin II, obesity, and insulin resistance stimulate specific brain areas such as the subfornical organ [117], hypothalamus, and rostral ventrolateral medulla [118], inducing oxidative stress and inflammation [119,120], which increases sympathetic outflow to promote vasoconstriction and salt retention, driving vascular and kidney damage. Interestingly, metabolic abnormalities particularly promote the sympathetic nervous system activity, which can further exacerbate metabolic dysfunction [121,122]. The interplay between the central nervous system and metabolic disorders can drive the sympathetic nervous system activity, inflammation, and oxidative stress, promoting hypertension and hypertensive organ damage. Interestingly, there are sex and race differences in these pathways. For example, sympathetic activity was elevated in obese white women with hypertension, and trimethaphan reduced blood pressure in hypertensive patients compared with those with normotension (−26.8 mm Hg versus −14.8 mm Hg) [123]. Meanwhile, there was no difference in the depressor responses induced by trimethaphan between obese black women with and without hypertension. It is suggested that premenopausal women are protected from obesity-induced metabolic complications; however, postmenopausal estrogen deprivation can promote sympathetic activity [124]. Metabolic disorders can affect multiple central nervous system pathways [121], particularly mitochondrial function [125]. Metabolic disorders play a critical role in hypertension and cardiovascular disease [126], this may include the central and peripheral role of mitochondria. Recent studies of brain mitochondrial proteome acetylation showed higher reliance on Sirt3-mediated deacetylation in females compared with males, and Sirt3 deficiency promoted behavioral changes [127]. It has been suggested that Sirt3 activation can be used for the treatment of neurodegenerative conditions [128]. We think that mitochondrial resilience is important for neurovascular and nervous systems, and new studies can provide novel mechanistic insight into the role of protein acetylation and support for new pharmacological targeting of mitochondria.

## Conclusion

In the present work, we have discussed different metabolic, oxidative stress, inflammatory, endocrine, genetic, vascular, and central pathways, with the emphasis on the role of mitochondria in these pathways contributing to the development of hypertension and cardiovascular disease. In the past two decades, we have made significant progress in understanding the molecular mechanisms of these pathological conditions, which now include a critical role of mitochondrial function. Meanwhile, the growing ‘epidemic’ of metabolic disease [129] combined with ∼50% prevalence of hypertension in the adult population [130] highlights the unmet need for new strategies to prevent, diagnose, and treat these conditions. Both hypertension and metabolic disease are linked to impaired fatty acid oxidation and oxidative stress associated with mitochondrial dysfunction; however, there are no clinically approved mitochondria-targeted treatments. We have a substantial gap of knowledge, and there is still a lot to be done. First, we must recognize an unmet need for treatment of pulmonary, cardiovascular, and kidney diseases, particularly in women. Women’s or female-specific risk factors are understudied in basic, clinical, and population research and hypertension guidelines. Sex-specific risk factors remained understudied in basic, clinical, and population research. Second, many basic science discoveries are lost in translation. We know that oxidative stress and mitochondrial dysfunction play an important role in cardiovascular disease; however, supplementation with common antioxidants like vitamin C and vitamin E is not effective, and mitochondria-targeted therapies are not available. We think that previously used antioxidants did not target the critical sources of oxidants such as mitochondria, and we are only beginning to understand how we should manipulate and normalize the acetylation of mitochondrial proteins, which is critical for mitochondrial health. Finally, we need to embrace the new paradigm for the critical role of fatty acid oxidation in human health rather than support ‘fat-free’ diets, and we need to invest in mitochondrial studies showing therapeutic promise for nutritional supplements and mitochondrial transplantation. Future translational studies must define the most important mitochondrial targets and effective therapeutic approaches to offset the pathological mechanisms responsible for impaired fatty acid oxidation, mitochondrial dysfunction, and oxidative stress.

## Open Access

Open access for this article was enabled by the participation of Vanderbilt University in an all-inclusive Read & Publish agreement with Portland Press and the Biochemical Society.

## Abbreviations

DAMPs: damage-associated molecular patterns
LCAD: long-chain acyl coenzyme A dehydrogenase
ROS: reactive oxygen species
SOD2: superoxide dismutase 2
Sirt3: sirtuin 3, mitochondrial deacetylase
GCN5L1: general control of amino acid synthesis 5 like 1
IsoLGs: isolevuglandins, highly reactive dialdehydes
ETC: electron transport chain
PUFA: polyunsaturated fatty acids

## References

[1] Cai H. and Harrison D.G. (2000) Endothelial dysfunction in cardiovascular diseases: the role of oxidant stress. Circ. Res. 87, 840–844 10.1161/01.RES.87.10.840 11073878

[2] Ahmad F.B. and Anderson R.N. (2021) The leading causes of death in the US for 2020. JAMA 325, 1829–1830 10.1001/jama.2021.5469 33787821 PMC8145781

[3] Harrison D.G., Marvar P.J. and Titze J.M. (2012) Vascular inflammatory cells in hypertension. Front. Physiol. 3, 128 10.3389/fphys.2012.00128 22586409 PMC3345946

[4] Tanaka A. and Node K. (2024) Associations of metabolic disorders with hypertension and cardiovascular disease: recent findings and therapeutic perspectives. Hypertens. Res. 47, 3338–3344 10.1038/s41440-024-01737-0 38811824

[5] Wenger N.K., Arnold A., Bairey Merz C.N., Cooper-DeHoff R.M., Ferdinand K.C., Fleg J.L. et al. (2018) Hypertension across a woman’s life cycle. J. Am. Coll. Cardiol. 71, 1797–1813 10.1016/j.jacc.2018.02.033 29673470 PMC6005390

[6] Ostchega Y., Fryar C.D., Nwankwo T. and Nguyen D.T. (2020) Hypertension prevalence among adults aged 18 and over: United States, 2017–2018. NCHS Data Brief. 1–8 32487290

[7] Chapman N., Ching S.M., Konradi A.O., Nuyt A.M., Khan T., Twumasi-Ankrah B. et al. (2023) Arterial hypertension in women: state of the art and knowledge gaps. Hypertension 80, 1140–1149 10.1161/HYPERTENSIONAHA.122.20448 36919603

[8] Wang X., Hao G., Chen L., Yang Y., Zhou H., Kang Y. et al. (2022) Hypertension-mediated organ damage and established cardiovascular disease in patients with hypertension: the China Hypertension Survey, 2012–2015. J. Hum. Hypertens. 36, 1092–1098 10.1038/s41371-021-00635-z 34799686 PMC9734033

[9] Harrison D.G. (2013) The Mosaic Theory revisited: common molecular mechanisms coordinating diverse organ and cellular events in hypertension. J. Am. Soc. Hypertens. 7, 68–74 10.1016/j.jash.2012.11.007 23321405 PMC3646628

[10] Dikalov S.I. and Ungvari Z. (2013) Role of mitochondrial oxidative stress in hypertension. Am. J. Physiol. Heart Circ. Physiol. 305, H1417–H1427 10.1152/ajpheart.00089.2013 24043248 PMC3840266

[11] Hirschey M.D., Shimazu T., Huang J.Y. and Verdin E. (2009) Acetylation of mitochondrial proteins. Methods Enzymol. 457, 137–147 10.1016/S0076-6879(09)05008-3 19426866

[12] Ghanta S., Grossmann R.E. and Brenner C. (2013) Mitochondrial protein acetylation as a cell-intrinsic, evolutionary driver of fat storage: chemical and metabolic logic of acetyl-lysine modifications. Crit. Rev. Biochem. Mol. Biol. 48, 561–574 10.3109/10409238.2013.838204 24050258 PMC4113336

[13] Dikalov S.I., Gutor S. and Dikalova A.E. (2023) Pathological mechanisms of cigarette smoking, dietary, and sedentary lifestyle risks in vascular dysfunction: mitochondria as a common target of risk factors. Pflügers Archiv. Eur. J. Physiol. 475, 857–866 10.1007/s00424-023-02806-y 36995495 PMC10911751

[14] Hosp F., Lassowskat I., Santoro V., De Vleesschauwer D., Fliegner D., Redestig H. et al. (2017) Lysine acetylation in mitochondria: From inventory to function. Mitochondrion 33, 58–71 10.1016/j.mito.2016.07.012 27476757

[15] Osborne B., Bentley N.L., Montgomery M.K. and Turner N. (2016) The role of mitochondrial sirtuins in health and disease. Free Radic. Biol. Med. 100, 164–174 10.1016/j.freeradbiomed.2016.04.197 27164052

[16] Kane A.E. and Sinclair D.A. (2018) Sirtuins and NAD(+) in the development and treatment of metabolic and cardiovascular diseases. Circ. Res. 123, 868–885 10.1161/CIRCRESAHA.118.312498 30355082 PMC6206880

[17] Vinten K.T., Tretowicz M.M., Coskun E., van Weeghel M., Canto C., Zapata-Perez R. et al. (2025) NAD(+) precursor supplementation in human ageing: clinical evidence and challenges. Nat. Metab. 7, 1974–1990 10.1038/s42255-025-01387-7 41083806

[18] Willis E.J. (1992) The powerhouse of the cell. Ultrastruct. Pathol. 16, iii–vi 10.3109/01913129209061353 1585494

[19] Lee W., Zamudio-Ochoa A., Buchel G., Podlesniy P., Marti Gutierrez N., Puigros M. et al. (2023) Molecular basis for maternal inheritance of human mitochondrial DNA. Nat. Genet. 55, 1632–1639 10.1038/s41588-023-01505-9 37723262 PMC10763495

[20] Chiaratti M.R., Garcia B.M., Carvalho K.F., Machado T.S., Ribeiro F. and Macabelli C.H. (2018) The role of mitochondria in the female germline: Implications to fertility and inheritance of mitochondrial diseases. Cell Biol. Int. 42, 711–724 10.1002/cbin.10947 29418047

[21] Kaltsas A., Moustakli E., Zikopoulos A., Georgiou I., Dimitriadis F., Symeonidis E.N. et al. (2023) Impact of advanced paternal age on fertility and risks of genetic disorders in offspring. Genes (Basel) 14, 486 10.3390/genes14020486 36833413 PMC9957550

[22] Vina J., Sastre J., Pallardo F. and Borras C. (2003) Mitochondrial theory of aging: importance to explain why females live longer than males. Antioxid. Redox Signal. 5, 549–556 10.1089/152308603770310194 14580309

[23] Sakamuri S.S., Sure V.N., Kolli L., Evans W.R., Sperling J.A., Bix G.J. et al. (2022) Aging related impairment of brain microvascular bioenergetics involves oxidative phosphorylation and glycolytic pathways. J. Cereb. Blood Flow Metab. 42, 1410–1424 10.1177/0271678X211069266 35296173 PMC9274865

[24] Dikalova A., Fehrenbach D., Mayorov V., Panov A., Ao M., Lantier L. et al. (2024) Mitochondrial CypD acetylation promotes endothelial dysfunction and hypertension. Circ. Res. 134, 1451–1464 10.1161/CIRCRESAHA.123.323596 38639088 PMC11116043

[25] Wilson C., Lee M.D., Buckley C., Zhang X. and McCarron J.G. (2023) Mitochondrial ATP production is required for endothelial cell control of vascular tone. Function (Oxf) 4, zqac063 10.1093/function/zqac063 36778749 PMC9909368

[26] Ibrahim A., Yucel N., Kim B. and Arany Z. (2020) Local mitochondrial ATP production regulates endothelial fatty acid uptake and transport. Cell Metab. 32, 309e307–319e307 10.1016/j.cmet.2020.05.018 32521232 PMC7415739

[27] Xiong J., Kawagishi H., Yan Y., Liu J., Wells Q.S., Edmunds L.R. et al. (2018) A metabolic basis for endothelial-to-mesenchymal transition. Mol. Cell 69, 689e7–698e7 10.1016/j.molcel.2018.01.010 29429925 PMC5816688

[28] Panov A. (2018) Synergistic oxidation of fatty acids, glucose and amino acids metabolites by isolated rat heart mitochondria. EC Cardiol. 5, 198–208 29532796

[29] Dikalov S., Panov A. and Dikalova A. (2024) Critical role of mitochondrial fatty acid metabolism in normal cell function and pathological conditions. Int. J. Mol. Sci. 25,6498 10.3390/ijms25126498 PMC11203650 38928204

[30] Panov A.V., Mayorov V.I., Dikalova A.E. and Dikalov S.I. (2022) Long-chain and medium-chain fatty acids in energy metabolism of murine kidney mitochondria. Int. J. Mol. Sci. 24,379 10.3390/ijms24010379 36613826 PMC9820327

[31] Cikic S., Chandra P.K., Harman J.C., Rutkai I., Katakam P.V., Guidry J.J. et al. (2021) Sexual differences in mitochondrial and related proteins in rat cerebral microvessels: a proteomic approach. J. Cereb. Blood Flow Metab. 41, 397–412 10.1177/0271678X20915127 32241204 PMC8370005

[32] Rutkai I., Merdzo I., Wunnava S., McNulty C., Chandra P.K., Katakam P.V. et al. (2022) Detrimental effects of transient cerebral ischemia on middle cerebral artery mitochondria in female rats. Am. J. Physiol. Heart Circ. Physiol. 323, H1343–H1351 10.1152/ajpheart.00346.2022 36367688 PMC9744641

[33] Sure V.N., Oruganti L., Sakamuri S., Pasupulati S.C., Ageeli R.Y., Chandra P. et al. (2026) Sex-dependent differences in bioenergetics of young mouse brain microvasculature: implications for oxygen-glucose deprivation and reoxygenation injury. Am. J. Physiol. Heart Circ. Physiol.330, H671-H685 10.1152/ajpheart.00195.2025 41525102 PMC12983451

[34] Montero D., Madsen K., Meinild-Lundby A.K., Edin F. and Lundby C. (2018) Sexual dimorphism of substrate utilization: differences in skeletal muscle mitochondrial volume density and function. Exp. Physiol. 103, 851–859 10.1113/EP087007 29626373

[35] Cano A., Ventura L., Martinez G., Cugusi L., Caria M., Deriu F. et al. (2022) Analysis of sex-based differences in energy substrate utilization during moderate-intensity aerobic exercise. Eur. J. Appl. Physiol. 122, 29–70 10.1007/s00421-021-04802-5 34550468 PMC8748379

[36] Oliveira P.J., Carvalho R.A., Portincasa P., Bonfrate L. and Sardao V.A. (2012) Fatty acid oxidation and cardiovascular risk during menopause: a mitochondrial connection? J. Lipids. 2012, 365798 10.1155/2012/365798 22496981 PMC3306973

[37] Palmisano B.T., Zhu L., Eckel R.H. and Stafford J.M. (2018) Sex differences in lipid and lipoprotein metabolism. Mol. Metab. 15, 45–55 10.1016/j.molmet.2018.05.008 29858147 PMC6066747

[38] Maher A.C., Akhtar M., Vockley J. and Tarnopolsky M.A. (2010) Women have higher protein content of beta-oxidation enzymes in skeletal muscle than men. PloS ONE 5, e12025 10.1371/journal.pone.0012025 20700461 PMC2917369

[39] Wang M., Zhu Z., He X., Dai S., Liu R. and Liu J. (2025) The crosstalk between mitochondrial dysfunction and fatty acid metabolism in heart failure: mechanisms and therapeutic strategies. Front. Pharmacol. 16, 1679085 10.3389/fphar.2025.1679085 41142255 PMC12545069

[40] Grenon S.M., Aguado-Zuniga J., Hatton J.P., Owens C.D., Conte M.S. and Hughes-Fulford M. (2012) Effects of fatty acids on endothelial cells: inflammation and monocyte adhesion. J. Surg. Res. 177, e35–e43 10.1016/j.jss.2012.04.010 22572621 PMC3756552

[41] Dabrowska E. and Narkiewicz K. (2023) Hypertension and dyslipidemia: the two partners in endothelium-related crime. Curr. Atheroscler. Rep. 25, 605–612 10.1007/s11883-023-01132-z 37594602 PMC10471742

[42] Li Y., Aziz Q., Anderson N., Ojake L. and Tinker A. (2020) Endothelial ATP-sensitive potassium channel protects against the development of hypertension and atherosclerosis. Hypertension 76, 776–784 10.1161/HYPERTENSIONAHA.120.15355 32654556 PMC7418932

[43] Klop B., Elte J.W. and Cabezas M.C. (2013) Dyslipidemia in obesity: mechanisms and potential targets. Nutrients 5, 1218–1240 10.3390/nu5041218 23584084 PMC3705344

[44] Bharathi S.S., Zhang Y., Mohsen A.W., Uppala R., Balasubramani M., Schreiber E. et al. (2013) Sirtuin 3 (SIRT3) protein regulates long-chain acyl-CoA dehydrogenase by deacetylating conserved lysines near the active site. J. Biol. Chem. 288, 33837–33847 10.1074/jbc.M113.510354 24121500 PMC3837126

[45] Dikalova A.E., Itani H.A., Nazarewicz R.R., McMaster W.G., Fessel J.P., Flynn C.R. et al. (2017) Sirt3 impairment and SOD2 hyperacetylation in vascular oxidative stress and hypertension. Circ. Res. 121, 664–774 10.1161/CIRCRESAHA.117.310933 PMC5562527 28684630

[46] Hirschey M.D., Shimazu T., Huang J.Y., Schwer B. and Verdin E. (2011) SIRT3 regulates mitochondrial protein acetylation and intermediary metabolism. Cold Spring Harb. Symp. Quant. Biol. 76, 267–277 10.1101/sqb.2011.76.010850 22114326

[47] Zhu Y., Park S.H., Ozden O., Kim H.S., Jiang H., Vassilopoulos A. et al. (2012) Exploring the electrostatic repulsion model in the role of Sirt3 in directing MnSOD acetylation status and enzymatic activity. Free Radic. Biol. Med. 53, 828–833 10.1016/j.freeradbiomed.2012.06.020 22732184 PMC3418453

[48] Hirschey M.D., Shimazu T., Goetzman E., Jing E., Schwer B., Lombard D.B. et al. (2010) SIRT3 regulates mitochondrial fatty-acid oxidation by reversible enzyme deacetylation. Nature 464, 121–125 10.1038/nature08778 20203611 PMC2841477

[49] Qiu X., Brown K., Hirschey M.D., Verdin E. and Chen D. (2010) Calorie restriction reduces oxidative stress by SIRT3-mediated SOD2 activation. Cell Metab. 12, 662–667 10.1016/j.cmet.2010.11.015 21109198

[50] Shi L. and Tu B.P. (2015) Acetyl-CoA and the regulation of metabolism: mechanisms and consequences. Curr. Opin. Cell Biol. 33, 125–131 10.1016/j.ceb.2015.02.003 25703630 PMC4380630

[51] Demarquoy J. and Le Borgne F. (2015) Crosstalk between mitochondria and peroxisomes. World J. Biol. Chem. 6, 301–309 10.4331/wjbc.v6.i4.301 26629313 PMC4657118

[52] Wanders R.J., Waterham H.R. and Ferdinandusse S. (2016) Metabolic interplay between peroxisomes and other subcellular organelles including mitochondria and the endoplasmic reticulum. Front. Cell. Dev. Biol. 3, 83 10.3389/fcell.2015.00083 26858947 PMC4729952

[53] Panov A.V., Mayorov V.I. and Dikalov S.I. (2024) Role of fatty acids β-oxidation in the metabolic interactions between organs. Int. J. Mol. Sci. 25,12740 10.3390/ijms252312740 PMC11641656 39684455

[54] Pougovkina O., te Brinke H., Ofman R., van Cruchten A.G., Kulik W. et al. (2014) Mitochondrial protein acetylation is driven by acetyl-CoA from fatty acid oxidation. Hum. Mol. Genet. 23, 3513–3522 10.1093/hmg/ddu059 24516071

[55] Sivanand S., Viney I. and Wellen K.E. (2018) Spatiotemporal control of acetyl-CoA metabolism in chromatin regulation. Trends Biochem. Sci. 43, 61–74 10.1016/j.tibs.2017.11.004 29174173 PMC5741483

[56] Dikalov S.I. and Dikalova A. (2019) Crosstalk between mitochondrial hyperacetylation and oxidative stress in vascular dysfunction and hypertension. Antioxid. Redox Signal. 31, 710–721 10.1089/ars.2018.7632 30618267 PMC6708267

[57] Dikalov S.I. and Dikalova A.E. (2016) Contribution of mitochondrial oxidative stress to hypertension. Curr. Opin. Nephrol. Hypertens. 25, 73–80 10.1097/MNH.0000000000000198 26717313 PMC4766975

[58] Touyz R.M. and Briones A.M. (2011) Reactive oxygen species and vascular biology: implications in human hypertension. Hypertens. Res. 34, 5–14 10.1038/hr.2010.201 20981034

[59] Harrison D.G., Gongora M.C., Guzik T.J. and Widder J. (2007) Oxidative stress and hypertension. J. Am. Soc. Hypertens. 1, 30–44 10.1016/j.jash.2006.11.006 20409831

[60] Zinkevich N.S. and Gutterman D.D. (2011) ROS-induced ROS release in vascular biology: redox-redox signaling. Am. J. Physiol. Heart Circ. Physiol. 301, H647–H653 10.1152/ajpheart.01271.2010 21685266 PMC3191081

[61] Dikalov S. (2011) Cross talk between mitochondria and NADPH oxidases. Free Radic. Biol. Med. 51, 1289–1301 10.1016/j.freeradbiomed.2011.06.033 21777669 PMC3163726

[62] Nazarewicz R.R., Dikalova A.E., Bikineyeva A. and Dikalov S.I. (2013) Nox2 as a potential target of mitochondrial superoxide and its role in endothelial oxidative stress. Am. J. Physiol. Heart Circ. Physiol. 305, H1131–H1140 10.1152/ajpheart.00063.2013 23955717 PMC3798790

[63] Dikalov S.I., Nazarewicz R.R., Bikineyeva A., Hilenski L., Lassegue B., Griendling K. et al. (2014) Nox2-induced production of mitochondrial superoxide in angiotensin II–mediated endothelial oxidative stress and hypertension. Antioxid. Redox Signal. 20, 281–294 10.1089/ars.2012.4918 24053613 PMC3887459

[64] Itani H.A., Dikalova A.E., McMaster W.G., Nazarewicz R.R., Bikineyeva A.T., Harrison D.G. et al. (2016) Mitochondrial cyclophilin D in vascular oxidative stress and hypertension. Hypertension 67, 1218–1227 10.1161/HYPERTENSIONAHA.115.07085 27067720 PMC4865418

[65] Jastroch M., Divakaruni A.S., Mookerjee S., Treberg J.R. and Brand M.D. (2010) Mitochondrial proton and electron leaks. Essays Biochem. 47, 53–67 10.1042/bse0470053 20533900 PMC3122475

[66] Nohl H., Gille L., Kozlov A. and Staniek K. (2003) Are mitochondria a spontaneous and permanent source of reactive oxygen species? Redox Rep. 8, 135–141 10.1179/135100003225001502 12935310

[67] Dikalova A.E., Pandey A.K., Xiao L., Arslanbaeva L., Sidorova T., Lopez M.G. et al. (2020) Mitochondrial deacetylase Sirt3 reduces vascular dysfunction and hypertension while Sirt3 depletion in essential hypertension is linked to vascular inflammation and oxidative stress. Circ. Res. 126, 439–452 10.1161/CIRCRESAHA.119.315767 31852393 PMC7035170

[68] Borras C., Sastre J., Garcia-Sala D., Lloret A., Pallardo F.V. and Vina J. (2003) Mitochondria from females exhibit higher antioxidant gene expression and lower oxidative damage than males. Free Radic. Biol. Med. 34, 546–552 10.1016/S0891-5849(02)01356-4 12614843

[69] Barris C.T., Faulkner J.L. and Belin de Chantemele E.J. (2023) Salt sensitivity of blood pressure in women. Hypertension 80, 268–278 10.1161/HYPERTENSIONAHA.122.17952 35997024 PMC9851945

[70] Dikalova A.E., Bikineyeva A.T., Budzyn K., Nazarewicz R.R., McCann L., Lewis W. et al. (2010) Therapeutic targeting of mitochondrial superoxide in hypertension. Circ. Res. 107, 106–116 10.1161/CIRCRESAHA.109.214601 20448215 PMC2901409

[71] Dikalova A.E., Kiriljuk I.A. and Dikalov S.I. (2015) Antihypertensive effect of mitochondria-targeted proxyl nitroxides. Redox Biol. 4, 301–312 10.1016/j.redox.2014.12.012 PMC4326181 25677087

[72] Parodi-Rullan R.M., Chapa-Dubocq X.R. and Javadov S. (2018) Acetylation of mitochondrial proteins in the heart: the role of SIRT3. Front. Physiol. 9, 1094 10.3389/fphys.2018.01094 30131726 PMC6090200

[73] Davies S.D., May-Zhang L.S., Boutaud O., Amarnath V., Kirabo A. and Harrison D.G. (2019) Isolevuglandins as mediators of disease and the development of dicarbonyl scavengers as pharmaceutical interventions. Pharmacol. Ther. 205, 107418 10.1016/j.pharmthera.2019.107418 31629006 PMC7495735

[74] Kirabo A., Fontana V., de Faria A.P., Loperena R., Galindo C.L., Wu J. et al. (2014) DC isoketal-modified proteins activate T cells and promote hypertension. J. Clin. Invest. 124, 4642–4656 10.1172/JCI74084 25244096 PMC4220659

[75] Trott D.W., Thabet S.R., Kirabo A., Saleh M.A., Itani H., Norlander A.E. et al. (2014) Oligoclonal CD8^+^ T cells play a critical role in the development of hypertension. Hypertension 64, 1108–1115 10.1161/HYPERTENSIONAHA.114.04147 25259750 PMC4191997

[76] de la Visitacion N., Chen W., Krishnan J., Van Beusecum J.P., Amarnath V., Hennen E.M. et al. (2024) Immunoproteasomal processing of IsoLG-adducted proteins is essential for hypertension. Circ. Res. 134, 1276–1291 10.1161/CIRCRESAHA.124.324068 38623763 PMC11081850

[77] Harrison D.G., Coffman T.M. and Wilcox C.S. (2021) Pathophysiology of hypertension: the mosaic theory and beyond. Circ. Res. 128, 847–863 10.1161/CIRCRESAHA.121.318082 33793328 PMC8023760

[78] McMaster W.G., Kirabo A., Madhur M.S. and Harrison D.G. (2015) Inflammation, immunity, and hypertensive end-organ damage. Circ. Res. 116, 1022–1033 10.1161/CIRCRESAHA.116.303697 25767287 PMC4535695

[79] Guzik T.J., Nosalski R., Maffia P. and Drummond G.R. (2024) Immune and inflammatory mechanisms in hypertension. Nat. Rev. Cardiol. 21, 396–416 10.1038/s41569-023-00964-1 38172242

[80] Yang S., Huang G. and Ting J.P. (2025) Mitochondria and NLRP3: to die or inflame. Immunity 58, 5–7 10.1016/j.immuni.2024.12.007 39813994 PMC11998340

[81] Heid M.E., Keyel P.A., Kamga C., Shiva S., Watkins S.C. and Salter R.D. (2013) Mitochondrial reactive oxygen species induces NLRP3-dependent lysosomal damage and inflammasome activation. J. Immunol. 191, 5230–5238 10.4049/jimmunol.1301490 24089192 PMC3833073

[82] Bloodworth N., Chen W., Hunter K., Patrick D., Palubinsky A., Phillips E. et al. (2024) Posttranslationally modified self-peptides promote hypertension in mouse models. J. Clin. Invest. 134, e174374 10.1172/JCI174374 39145457 PMC11324298

[83] Li W., Cao X., Wang S., Jin X. and Wang H. (2025) GCN5L1 aggravates postherpetic neuralgia through regulating microglial mitochondrial fission-fusion homeostasis. J. Cell. Mol. Med. 29, e70861 10.1111/jcmm.70861 40988108 PMC12457213

[84] Kurundkar D., Kurundkar A.R., Bone N.B., Becker E.J. Jr., Liu W., Chacko B. et al. (2019) SIRT3 diminishes inflammation and mitigates endotoxin-induced acute lung injury. JCI Insight 4, e120722 10.1172/jci.insight.120722 30626741 PMC6485358

[85] Dikalova A., Ao M., Tkachuk L. and Dikalov S. (2024) Deacetylation mimetic mutation of mitochondrial SOD2 attenuates ANG II-induced hypertension by protecting against oxidative stress and inflammation. Am. J. Physiol. Heart Circ. Physiol. 327, H433–H443 10.1152/ajpheart.00162.2024 38904850 PMC11442025

[86] Morgan M.J. and Liu Z.G. (2011) Crosstalk of reactive oxygen species and NF-kappaB signaling. Cell Res. 21, 103–115 10.1038/cr.2010.178 21187859 PMC3193400

[87] Gurung P., Lukens J.R. and Kanneganti T.D. (2015) Mitochondria: diversity in the regulation of the NLRP3 inflammasome. Trends Mol. Med. 21, 193–201 10.1016/j.molmed.2014.11.008 25500014 PMC4352396

[88] Klinge C.M. (2020) Estrogenic control of mitochondrial function. Redox Biol. 31, 101435 10.1016/j.redox.2020.101435 32001259 PMC7212490

[89] Ko S.H. and Jung Y. (2021) Energy metabolism changes and dysregulated lipid metabolism in postmenopausal women. Nutrients 13, 4556 34960109 10.3390/nu13124556 PMC8704126 34960109

[90] Visniauskas B., Kilanowski-Doroh I., Ogola B.O., McNally A.B., Horton A.C., Imulinde Sugi A. et al. (2023) Estrogen-mediated mechanisms in hypertension and other cardiovascular diseases. J. Hum. Hypertens. 37, 609–618 10.1038/s41371-022-00771-0 36319856 PMC10919324

[91] Lejri I., Grimm A. and Eckert A. (2018) Mitochondria, estrogen and female brain aging. Front. Aging Neurosci. 10, 124 10.3389/fnagi.2018.00124 29755342 PMC5934418

[92] Nwia S.M., Leite A.P.O., Li X.C. and Zhuo J.L. (2023) Sex differences in the renin-angiotensin-aldosterone system and its roles in hypertension, cardiovascular, and kidney diseases. Front. Cardiovasc. Med. 10, 1198090 10.3389/fcvm.2023.1198090 37404743 PMC10315499

[93] Mirabito K.M., Hilliard L.M., Head G.A., Widdop R.E. and Denton K.M. (2014) Pressor responsiveness to angiotensin II in female mice is enhanced with age: role of the angiotensin type 2 receptor. Biol. Sex Differ. 5, 13 10.1186/s13293-014-0013-7 25774285 PMC4358320

[94] Barsha G., Mirabito Colafella K.M., Walton S.L., Gaspari T.A., Spizzo I., Pinar A.A. et al. (2021) In aged females, the enhanced pressor response to angiotensin ii is attenuated by estrogen replacement via an angiotensin type 2 receptor-mediated mechanism. Hypertension 78, 128–137 10.1161/HYPERTENSIONAHA.121.17164 33966450

[95] Rogers J.L., Mitchell A.R., Maric C., Sandberg K., Myers A. and Mulroney S.E. (2007) Effect of sex hormones on renal estrogen and angiotensin type 1 receptors in female and male rats. Am. J. Physiol. Regul. Integr. Comp. Physiol. 292, R794–R799 10.1152/ajpregu.00424.2006 16990489

[96] Escobales N., Nunez R.E. and Javadov S. (2019) Mitochondrial angiotensin receptors and cardioprotective pathways. Am. J. Physiol. Heart Circ. Physiol. 316, H1426–H1438 10.1152/ajpheart.00772.2018 30978131 PMC6620675

[97] Seidel E. and Scholl U.I. (2017) Genetic mechanisms of human hypertension and their implications for blood pressure physiology. Physiol. Genomics 49, 630–652 10.1152/physiolgenomics.00032.2017 28887369

[98] Manosroi W. and Williams G.H. (2019) Genetics of human primary hypertension: focus on hormonal mechanisms. Endocr. Rev. 40, 825–856 10.1210/er.2018-00071 30590482 PMC6936319

[99] Nurkkala J., Vaura F., Toivonen J. and Niiranen T. (2024) Genetics of hypertension-related sex differences and hypertensive disorders of pregnancy. Blood Press. 33, 2408574 10.1080/08037051.2024.2408574 39371034

[100] Laporte M.A.L. and Coutinho T. (2024) Vascular aging in women. Can. J. Cardiol. 40, 1493–1495 10.1016/j.cjca.2024.01.036 38325763

[101] Wise I.A. and Charchar F.J. (2016) Epigenetic modifications in essential hypertension. Int. J. Mol. Sci. 17, 451 10.3390/ijms17040451 27023534 PMC4848907

[102] Mani A. (2024) Update in genetic and epigenetic causes of hypertension. Cell. Mol. Life Sci. 81, 201 10.1007/s00018-024-05220-4 38691164 PMC11062952

[103] Tuscher J.J. and Day J.J. (2019) Multigenerational epigenetic inheritance: one step forward, two generations back. Neurobiol. Dis. 132, 104591 10.1016/j.nbd.2019.104591 31470104

[104] Santos J.H. (2021) Mitochondria signaling to the epigenome: a novel role for an old organelle. Free Radic. Biol. Med. 170, 59–69 10.1016/j.freeradbiomed.2020.11.016 33271282 PMC8166959

[105] Mani S., Srivastava V., Shandilya C., Kaushik A. and Singh K.K. (2024) Mitochondria: the epigenetic regulators of ovarian aging and longevity. Front. Endocrinol. (Lausanne) 15, 1424826 10.3389/fendo.2024.1424826 39605943 PMC11598335

[106] Ostaiza-Cardenas J., Tobar A.C., Costa S.C., Calero D.S., Lopez-Carrera A., Bermudez F.G. et al. (2025) Epigenetic modulation by life-style: advances in diet, exercise, and mindfulness for disease prevention and health optimization. Front. Nutr. 12, 1632999 10.3389/fnut.2025.1632999 40917096 PMC12408607

[107] Betai D., Ahmed A.S., Saxena P., Rashid H., Patel H., Shahzadi A. et al. (2024) Gender disparities in cardiovascular disease and their management: a review. Cureus 16, e59663 10.7759/cureus.59663 38836150 PMC11148660

[108] Kalenga C.Z., Metcalfe A., Robert M., Nerenberg K.A., MacRae J.M. and Ahmed S.B. (2023) Association between the route of administration and formulation of estrogen therapy and hypertension risk in postmenopausal women: a prospective population-based study. Hypertension 80, 1463–1473 10.1161/HYPERTENSIONAHA.122.19938 37272379

[109] Tian J., Yi L., Lu Y., Kou Y., Zhang L., Jin P. et al. (2026) Mitochondrial dysfunction in hypertension: mechanistic pathways and therapeutic implications. Funct. Integr. Genomics 26, 40 10.1007/s10142-025-01813-9 41656396

[110] Leung S.W.S. and Shi Y. (2022) The glycolytic process in endothelial cells and its implications. Acta Pharmacol. Sin. 43, 251–259 10.1038/s41401-021-00647-y 33850277 PMC8791959

[111] Archer S.L. (2017) Pyruvate kinase and warburg metabolism in pulmonary arterial hypertension: uncoupled glycolysis and the cancer-like phenotype of pulmonary arterial hypertension. Circulation 136, 2486–2490 10.1161/CIRCULATIONAHA.117.031655 29255124 PMC5739072

[112] Dikalova A., Ao M., Lantier L., Gutor S. and Dikalov S. (2025) Depletion of mitochondrial CypD in endothelial and smooth muscle cells attenuates vascular dysfunction and hypertension. Function (Oxf) 6, zqaf006 10.1093/function/zqaf006 39919759 PMC11931617

[113] Torimoto K., Okuno K., Kuroda R., Shanas N., Cicalese S.M., Eguchi K. et al. (2022) Glucose consumption of vascular cell types in culture: toward optimization of experimental conditions. Am. J. Physiol. Cell Physiol. 322, C73–C85 10.1152/ajpcell.00257.2021 34817269 PMC8791793

[114] Oller J., Gabande-Rodriguez E., Ruiz-Rodriguez M.J., Desdin-Mico G., Aranda J.F., Rodrigues-Diez R. et al. (2021) Extracellular tuning of mitochondrial respiration leads to aortic aneurysm. Circulation 143, 2091–2109 10.1161/CIRCULATIONAHA.120.051171 33709773 PMC8140666

[115] Tsuruda T., Hatakeyama K., Nagamachi S., Sekita Y., Sakamoto S., Endo G.J. et al. (2012) Inhibition of development of abdominal aortic aneurysm by glycolysis restriction. Arterioscler. Thromb. Vasc. Biol. 32, 1410–1417 10.1161/ATVBAHA.111.237065 22499992

[116] Oller J., Gabande-Rodriguez E., Roldan-Montero R., Ruiz-Rodriguez M.J., Redondo J.M., Martin-Ventura J.L. et al. (2022) Rewiring vascular metabolism prevents sudden death due to aortic ruptures-brief report. Arterioscler. Thromb. Vasc. Biol. 42, 462–469 10.1161/ATVBAHA.121.317346 35196876

[117] Lob H.E., Schultz D., Marvar P.J., Davisson R.L. and Harrison D.G. (2013) Role of the NADPH oxidases in the subfornical organ in angiotensin II-induced hypertension. Hypertension 61, 382–387 10.1161/HYPERTENSIONAHA.111.00546 23248154 PMC3678909

[118] Braga V.A., Medeiros I.A., Ribeiro T.P., Franca-Silva M.S., Botelho-Ono M.S. and Guimaraes D.D. (2011) Angiotensin-II-induced reactive oxygen species along the SFO-PVN-RVLM pathway: implications in neurogenic hypertension. Braz. J. Med. Biol. Res. 44, 871–876 10.1590/S0100-879X2011007500088 21755262

[119] Lob H.E., Marvar P.J., Guzik T.J., Sharma S., McCann L.A., Weyand C. et al. (2010) Induction of hypertension and peripheral inflammation by reduction of extracellular superoxide dismutase in the central nervous system. Hypertension 55, 277–283 10.1161/HYPERTENSIONAHA.109.142646 20008675 PMC2813894

[120] Marvar P.J., Vinh A., Thabet S., Lob H.E., Geem D., Ressler K.J. et al. (2012) T lymphocytes and vascular inflammation contribute to stress-dependent hypertension. Biol. Psychiatry 71, 774–782 10.1016/j.biopsych.2012.01.017 22361077 PMC3354001

[121] Rojas M., Chavez-Castillo M., Pirela D., Parra H., Nava M., Chacin M. et al. (2021) Metabolic syndrome: is it time to add the central nervous system? Nutrients 13, 2254 34208833 10.3390/nu13072254 PMC8308252 34208833

[122] Tanaka M. and Itoh H. (2019) Hypertension as a metabolic disorder and the novel role of the gut. Curr. Hypertens. Rep. 21, 63 10.1007/s11906-019-0964-5 31236708 PMC6591187

[123] Marinos A., Gamboa A., Celedonio J.E., Preheim B.A., Okamoto L.E., Ramirez C.E. et al. (2017) Hypertension in obese black women is not caused by increased sympathetic vascular tone. J. Am. Heart Assoc. 6, e006971 10.1161/JAHA.117.006971 29151035 PMC5721777

[124] Yoo J.K. and Fu Q. (2020) Impact of sex and age on metabolism, sympathetic activity, and hypertension. FASEB J. 34, 11337–11346 10.1096/fj.202001006RR 32779294

[125] Tripathi K. and Ben-Shachar D. (2024) Mitochondria in the central nervous system in health and disease: the puzzle of the therapeutic potential of mitochondrial transplantation. Cells 13, 410 38474374 10.3390/cells13050410 PMC10930936 38474374

[126] Chakraborty S., Mandal J., Yang T., Cheng X., Yeo J.Y., McCarthy C.G. et al. (2020) Metabolites and hypertension: insights into hypertension as a metabolic disorder: 2019 Harriet Dustan Award. Hypertension 75, 1386–1396 10.1161/HYPERTENSIONAHA.120.13896 32336227 PMC7225070

[127] Sidorova-Darmos E., Fallah M.S., Logan R., Lin C.Y. and Eubanks J.H. (2022) Mitochondrial brain proteome acetylation levels and behavioural responsiveness to amphetamine are altered in mice lacking Sirt3. Front. Physiol. 13, 948387 10.3389/fphys.2022.948387 36148309 PMC9489219

[128] Tyagi A. and Pugazhenthi S. (2023) A promising strategy to treat neurodegenerative diseases by SIRT3 activation. Int. J. Mol. Sci. 24, 1615 36675125 10.3390/ijms24021615 PMC9866791 36675125

[129] Liang X., Or B., Tsoi M.F., Cheung C.L. and Cheung B.M.Y. (2023) Prevalence of metabolic syndrome in the United States National Health and Nutrition Examination Survey 2011–18. Postgrad. Med. J. 99, 985–992 10.1093/postmj/qgad008 36906842

[130] Byrd J.B., Zeng C., Tavel H.M., Magid D.J., O'Connor P.J., Margolis K.L. et al. (2011) Combination therapy as initial treatment for newly diagnosed hypertension. Am. Heart J. 162, 340–346 10.1016/j.ahj.2011.05.010 21835296 PMC3153357

